# Effect modification of tumor necrosis factor-α on the kynurenine and serotonin pathways in major depressive disorder on type 2 diabetes mellitus

**DOI:** 10.1007/s00406-023-01713-8

**Published:** 2023-11-22

**Authors:** Naomichi Okamoto, Takashi Hoshikawa, Yuichi Honma, Enkhmurun Chibaatar, Atsuko Ikenouchi, Masaru Harada, Reiji Yoshimura

**Affiliations:** 1 Department of Psychiatry, University of Occupational and Environmental Health, 807-8555, Kitakyushu, Fukuoka, 8078555 Japan; 2 Third Department of Internal Medicine, University of Occupational and Environmental Health, Fukuoka, Japan; 3 Medical Center for Dementia, University Hospital, University of Occupational and Environmental Health, Fukuoka, Japan

**Keywords:** Major depressive disorder, Type 2 diabetes mellitus, Kynurenine pathway, Tryptophan metabolism, Inflammatory cytokine, Tumor necrosis factor-α

## Abstract

Major depressive disorder (MDD) is strongly associated with type 2 diabetes mellitus (T2DM). The kynurenine and serotonin pathways, as well as chronic low-grade inflammation, are being considered potential links between them. MDD associated with T2DM is less responsive to treatment than that without T2DM; however, the underlying mechanism remains unknown. We aimed to investigate the effects of inflammatory cytokines on the kynurenine and serotonin pathways in patients with comorbid MDD and T2DM and those with only MDD. We recruited 13 patients with comorbid MDD and T2DM and 27 patients with only MDD. We measured interleukin-6 and tumor necrosis factor-α (TNF-α) levels as inflammatory cytokines and metabolites of the kynurenine pathway and examined the relationship between the two. TNF-α levels were significantly higher in patients with comorbid MDD and T2DM than in those with only MDD in univariate (p = 0.044) and multivariate (adjusted p = 0.036) analyses. TNF-α showed a statistically significant effect modification (interaction) with quinolinic acid/tryptophan and serotonin in patients from both groups (β = 1.029, adjusted p < 0.001; β =  − 1.444, adjusted p = 0.047, respectively). Limitations attributed to the study design and number of samples may be present. All patients were Japanese with mild to moderate MDD; therefore, the generalizability of our findings may be limited. MDD with T2DM has more inflammatory depression components and activations of the kynurenine pathway by inflammatory cytokines than MDD without T2DM. Hence, administering antidepressants and anti-inflammatory drugs in combination may be more effective in patients with comorbid MDD and T2DM.

**Supplementary Information:**

The online version contains supplementary material available at 10.1007/s00406-023-01713-8.

## Introduction

Major depressive disorder (MDD) and type 2 diabetes mellitus (T2DM) are common diseases worldwide, and both have significant effects on mental and physical health [1, 2]. Although the pathophysiologies of MDD and T2DM remain unknown, several studies have demonstrated a strong association between MDD and T2DM [3–5]. MDD increases the risk of metabolism and metabolic diseases, such as T2DM and obesity [6]; additionally, depressive symptoms and MDD frequently occur in patients with T2DM [7].

Direct or indirect dysregulation of the kynurenine and serotonin pathways [8–10] and chronic low-grade inflammation [11–13] (i.e., where inflammatory cytokines are chronically produced) have become increasingly important in recent years as metabolic pathways that link MDD and T2DM. The kynurenine and serotonin pathways involve the metabolism of tryptophan, which is an essential amino acid. Tryptophan is involved in serotonin synthesis in the serotonin pathway and is broken down from kynurenine to quinolinic acid through the kynurenine pathway, ultimately producing nicotinamide adenine dinucleotide (NAD) as an important cellular energy source [8].

Several studies have shown a relationship between MDD and the kynurenine and serotonin pathways [8, 14]; moreover, a previous meta-analysis has indicated increased levels of quinolinic acid in patients with MDD [15]. Previous studies have supported the involvement of kynurenine in the pathogenesis of T2DM [16] and regulation of glucose metabolism in vitro and in vivo [10]. Patients with T2DM have increased tryptophan metabolism (similar to patients with MDD) and downstream metabolite levels, and decreased tryptophan levels [17].

**Fig. 1:**
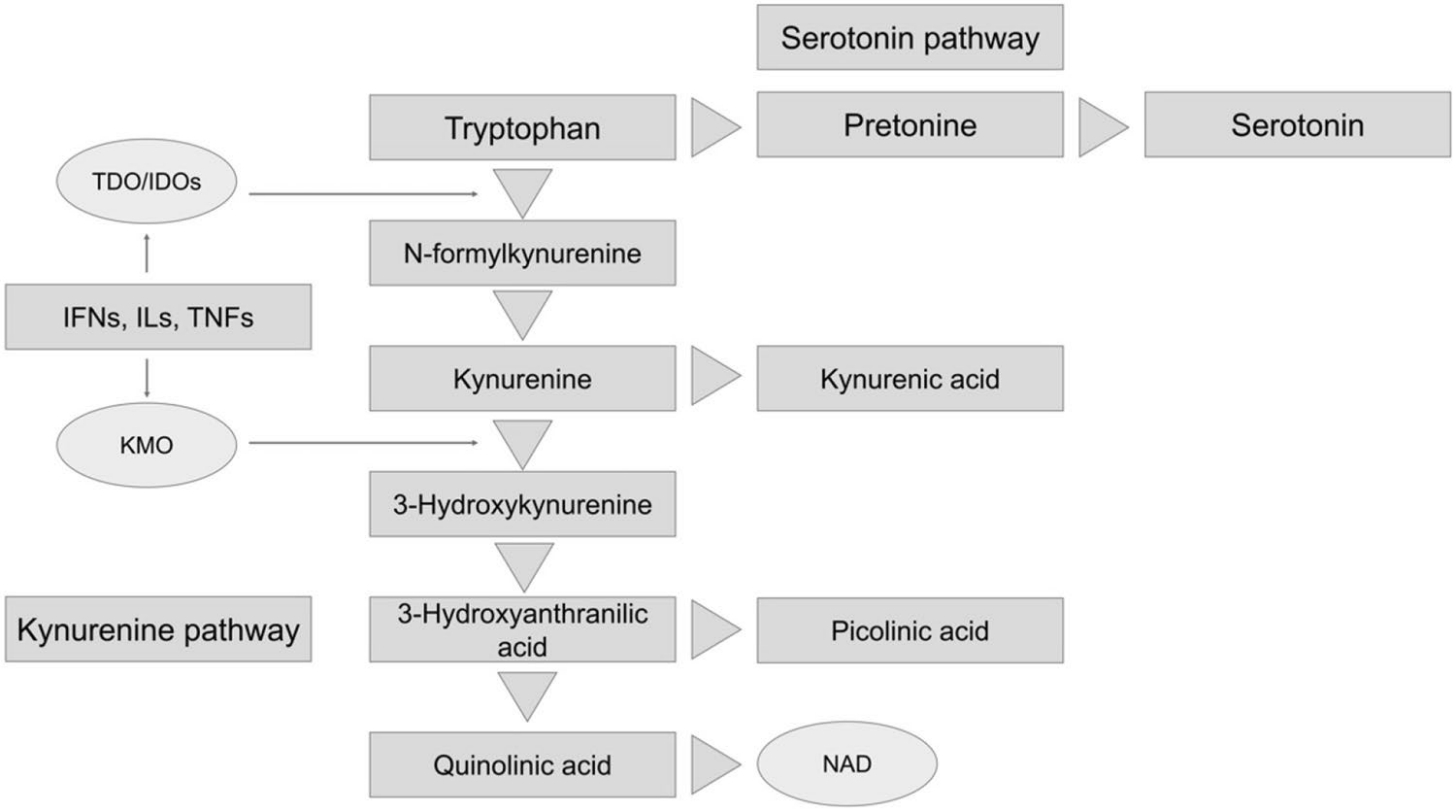
The kynurenine and serotonin pathways (tryptophan metabolism). The kynurenine and serotonin pathways are involved in the metabolism of tryptophan, which is an essential amino acid. Tryptophan is involved in serotonin synthesis and is broken down from kynurenine to quinolinic acid through the kynurenine pathway, ultimately producing nicotinamide adenine dinucleotide, a cellular energy source. *IFNs* interferons, *ILs* interleukins, *KMO* kynurenine 3-monooxygenase, *TDO/IDO* tryptophan 2,3-dioxygenase/indoleamine 2,3-dioxygenase, *TNFs* tumor necrosis factors, *NAD* nicotinamide adenine dinucleotide

Inflammatory cytokines redirect tryptophan metabolism toward kynurenine and quinolinic acid by upregulating tryptophan 2,3-dioxygenase/indoleamine 2,3-dioxygenase (TDO/IDO) and kynurenine 3-monooxygenase (Fig. 1) [8]. Inflammatory cytokines, such as interferons (IFNs), interleukins (ILs), and tumor necrosis factors (TNFs), are synergistically activated [18–22]. As activated immune cells are dependent on high levels of NAD, the shunt shift takes place to replenish these cells by increasing their metabolism to quinolinic acid [8]. Thus, in patients with MDD and T2DM in a chronic inflammatory state, shunt enhancement of the kynurenine and serotonin pathways is thought to chronically occur in the direction of the kynurenine pathway [8].

MDD associated with T2DM is less responsive to treatment than MDD without T2DM [23]. In recent years, inflammation has been involved in treatment-resistant MDD and has resulted in high inflammatory markers relative to those whose MDD is treatment-responsive [24, 25]; however, its pathophysiological significance is unknown. Additionally, differences in the effects of inflammatory cytokines on the kynurenine and serotonin pathways in patients with comorbid MDD and T2DM and those with MDD only remain unclear. Moreover, there are few reports on the differential effects of different types of inflammatory cytokines on the kynurenine and serotonin metabolic pathways.

We previously reported that comorbid T2DM affects overall metabolic pathways and alters the distribution of serum metabolites in patients with MDD [26]. In this study, we researched the effects of inflammatory cytokines on the common biological processes of MDD and T2DM, which have been shown to involve the kynurenine and serotonin pathways, as well as the clinical data of the patients. Specifically, we investigated three points: (1) whether the effects of inflammatory cytokines on the kynurenine and serotonin pathways in patients with MDD differ depending on whether they have T2DM or not (e.g., there is an effect modification of inflammatory cytokines on the metabolites of the kynurenine and serotonin pathways in MDD depending on comorbid T2DM); (2) whether different types of inflammatory cytokines have different effects on the kynurenine and serotonin pathways; and (3) whether there is a relationship between inflammatory cytokines and blood metabolites of the kynurenine and serotonin pathways and the clinical data of patients, such as psychiatric symptoms and laboratory data.

## Methods

### Ethics statement

This study complied with the 1975 Declaration of Helsinki (revised in 2008) and the Japanese Ethical Guidelines for Medical and Health Research Involving Human Subjects. All procedures involving human participants were approved by the Ethics Committee of the University of Occupational and Environmental Health, Japan (approval number: UOEHCRB21-057). Written informed consent was obtained from all the participants. To protect patient privacy, anonymous identification numbers were assigned to each patient.

### Participants

We recruited 40 patients with MDD diagnosed according to the Diagnostic and Statistical Manual of Mental Disorders, Fifth Edition from the Hospital of the University of Occupational and Environmental Health in Japan [27]. No patient had psychiatric comorbidities other than MDD. The exclusion criteria included a history of major neurological disease, epilepsy, cerebrovascular accident, head trauma with cognitive sequelae, and intellectual disability. T2DM was diagnosed according to the relevant Japan Diabetes Society criteria (i.e., fasting blood glucose levels ≥ 126 mg/dL and hemoglobin A1c (HbA1c) levels ≥ 6.5%); alternatively, if a patient was currently taking diabetes medication, then they were diagnosed accordingly. Psychiatrists and internists diagnosed MDD and T2DM respectively.

### Clinical assessment and blood sampling

We assessed the clinical symptoms of patients with MDD using the Hamilton Depression Rating Scale (HAMD) [28]. Non-fasting blood samples were collected, and serum samples were randomly collected. Blood samples were collected in plain blood tubes at the University of Occupational and Environmental Health Japan, separated by centrifugation at 2000 × g for 20 min, and stored at –80 °C in silicone-coated tubes until analysis.

### Measurement of inflammatory cytokines

TNF-α and IL-6levels were determined by SRL Inc. (Kitakyushu, Japan) using a sandwich enzyme immunoassay. The microplate was coated with a human monoclonal antibody as a capture antibody. Standards and samples were subsequently added to the wells, allowing the immobilized antibody to bind to any TNF-α and IL-6 present in the samples. After thorough washing to remove any unbound substances, a biotinylated polyclonal antibody specific to humans was introduced to the wells. After another round of washing to remove any unbound antibody-biotin reagent, an enzyme-linked streptavidin was added. Subsequently, any unbound streptavidin-enzyme reagent was washed away, and a substrate solution was added to initiate a color reaction that was directly proportional to the amount of TNF-α and IL-6 initially bound. The color development process was terminated, and the intensity of the resulting color was measured.

### Measurement of kynurenine and serotonin pathway metabolites

We performed metabolomics analysis to measure tryptophan metabolites. To perform the analysis, serum samples were sent to Human Metabolome Technologies, Inc. (HMT, Tsuruoka, Japan). Metabolites were measured using capillary electrophoresis (CE) coupled with Fourier transform mass spectrometry (CE-FTMS), following the procedure outlined in HMT’s ω Scan package. The CE-FTMS analysis was performed using specific equipment and software. An Agilent 7,100 CE system equipped with a Q Exactive Plus mass spectrometer (Thermo Fisher Scientific, Inc., Waltham, MA, USA) was used. The system also included an Agilent 1,260 isocratic HPLC pump, Agilent G1603A CE-MS adapter kit, and Agilent G1607A CE–ESI–MS sprayer kit (Agilent Technologies Inc., Santa Clara, CA, USA). The operation and control of these components were performed using the Agilent Mass Hunter workstation software version B.08.00 for the 6,200 series TOF/6,500 series Q-TOF (Agilent Technologies Inc., Santa Clara, CA, USA) and Xcalibur software (Thermo Fisher Scientific, Inc). A fused silica capillary with an internal diameter of 50 μm and a total length of 80 cm was used for the connections.

Commercial electrolytes from HMT were used as electrophoresis buffers, specifically H3301-1001 for cation analysis and I3302-1023 for anion analysis. The spectrometer scanned a mass range of 50–1,000. Peaks were analyzed using the Master Hands automatic integration software (Keio University, Tsuruoka, Yamagata, Japan) to obtain m/z, peak area, and migration time (MT) data. Signal peaks corresponding to isotopomers, adduct ions, and other product ions of known metabolites were excluded, and the remaining peaks were annotated according to the HMT metabolome database based on their m/z values and MTs. The areas of the annotated peaks were subsequently normalized to those of the internal standards and sample volumes to obtain the relative levels of each metabolite of the kynurenine pathway. Among these metabolites, we extracted the relative levels of tryptophan, *N*-formylkynurenine, kynurenine, kynurenic acid, 3-hydroxykynurenine, 3-hydroxyanthranilic acid, quinolinic acid, picolinic acid, xanthurenic acid, pretonine, serotonin, and N-methylserotonin.

### Data analysis

All statistical analyses were performed using Stata 17 (Stata Corp., College Station, TX, USA). Figures were created using Python 3.0 (Python Software Foundation, Wilmington, DE, USA). The normality of the data was assessed using histograms. In descriptive statistics, demographic data were expressed as the mean (standard deviation) or median [interquartile range] based on normality. The Mann–Whitney U test and multiple regression analysis were used to test for differences between the two groups. Spearman’s rank-sum test and multiple regression analysis were performed for each group to test for correlation. An interaction analysis was also performed by multiple regression analysis to assess for correlations that were statistically significant for one of the groups in the multiple regression analysis.

Interaction analysis between the effects of inflammatory cytokines on the kynurenine and serotonin pathways were measured by comparing the differences in the slopes of each regression line. The multiple regression analysis was adjusted for potential covariates including age, sex, and body mass index (BMI). The choice of covariates was based on sample size considerations and the three basic demographic datasets, which were preselected prior to performing the analysis. Missing data were excluded from the analysis. To avoid the influence of outliers, all data are expressed as medians [interquartile ranges] in the inferential statistics section, and nonparametric tests were selected. Multiple regression analysis and figure creation were performed after removing outliers using scatterplots, which were approximately two times the interquartile range. The validity of the model of multiple regression analysis was assessed by the normality of the residual histograms. P-values were calculated using two-tailed tests, and p < 0.05 was considered statistically significant.

## Results

### Demographic and clinical data

The demographic and clinical data of the patients with MDD are shown in Table 1. Overall, the severity of the depressive symptoms was mild to moderate. Among the 40 patients with MDD, 13 had T2DM, whereas 27 did not. Patients with comorbid MDD and T2DM were older and had higher overweight rates than those with only MDD. Among the 40 patients, 37 patients took antidepressant medications whereas the remaining three did not.

**Table 1:** Demographic and clinical characteristics of the patients

	MDD without T2DM (n = 27)	MDD with T2DM (n = 13)
Demographic		
Age, years	52.3 (13.9)	65.6 (13.1)
Sex, male	11 (39%)	6 (46%)
BMI, kg/m^2^	22.9 (5.33)	26.1 (6.18)
Family history of MDD	5 (18.5%)	2 (15.3%)
Family history of T2DM	7 (25.9%)	5 (38.4%)
Clinical data		
HAMD, points	16.2 (6.82)	13 (6.60)
Disease period, years	3 [0–5]	2 [1–19]
Past depressive episode, number	2 [1, 2]	2 [1, 2]
HbA1c, %	5.54 (0.47)	7.01 (1.17)

*BMI* body mass index, *HAMD* Hamilton Depression Rating Scale, *HbA1c* hemoglobin A1c, *MDD* major depressive disorder, *T2DM* type 2 diabetes mellitus

### Differences in inflammatory cytokine levels between both groups

**Fig. 2:**
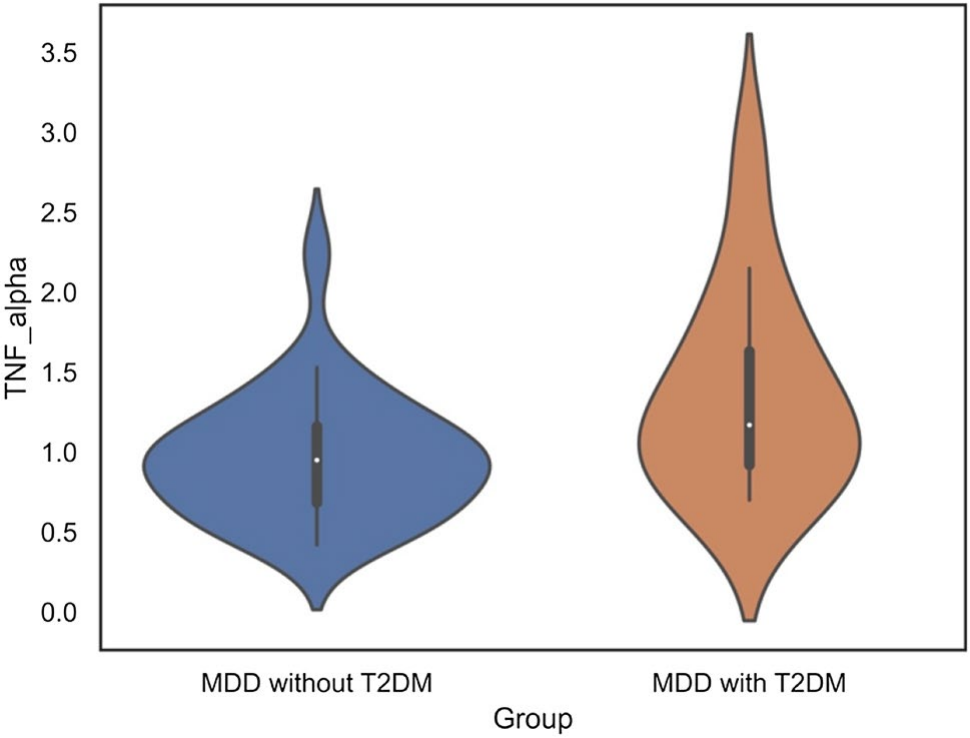
Differences in TNF-α levels between both groups. Violin plot showing that TNF-α levels are significantly higher in patients with MDD and T2DM than in those with only MDD. *TNF-α* tumor necrosis factor-α (pg/mL), *IL-6* interleukin-6 (pg/mL), *MDD* major depressive disorder, *T2DM* type 2 diabetes mellitus

Differences in inflammatory cytokine levels between patients with MDD and T2DM and those with only MDD are shown in the Table 2 and Fig. 2. There were no missing data in regard to TNF-α and IL-6. TNF-α levels were significantly higher in patients with MDD and T2DM [1.17 (0.92–1.63) pg/mL] than in those with only MDD [0.95 (0.68–1.16) pg/mL] in univariate (p = 0.044) and multivariate (adjusted p = 0.036) analyses. IL-6 showed no significant difference between patients with MDD and T2DM [1.3 (1.0–2.2) pg/mL] and those with only MDD [2.3 (1.4–3.9) pg/mL] (p = 0.068, adjusted p = 0.13).

**Table 2:** Differences in inflammatory cytokines between patients with major depressive disorder with and without type 2 diabetes mellitus

	MDD without T2DM	MDD with T2DM	p-value	Adjusted p-value	Effect size
TNF-α, pg/mL	0.95 [0.68–1.16]	1.17 [0.92–1.63]	0.044	0.036	0.32
IL-6, pg/mL	1.3 [1.0–2.2]	2.3 [1.4–3.9]	0.068	0.13	0.29

P-values are adjusted for age, sex, and BMI. The p-value was calculated using the Mann–Whitney U test, and the adjusted p-value was determined by multiple regression analysis. Effect sizes were calculated using the Mann–Whitney U test. *BMI* body mass index, *MDD* major depressive disorder, *T2DM* type 2 diabetes mellitus, *TNF-α* tumor necrosis factor-α, *IL-6* interleukin-6

### Differences in metabolites of the kynurenine and serotonin pathways and ratio between both groups

Differences in the metabolites of the kynurenine and serotonin pathways are shown in Tables 3, 4. Two samples of pretonine (all in patients with MDD and T2DM) and three samples of serotonin (two samples in MDD without T2DM and one sample in MDD with T2DM) had missing values. Kynurenic acid, 3-hydroxyanthranilic acid, picolinic acid, xanthurenic acid, and *N*-methylserotonin levels were below the detection level or insufficient to perform statistical analysis owing to the small sample size. The ratio of quinolinic acid/tryptophan was statistically higher in patients with MDD and T2DM [7.093 × 10^−4^ (5.442 × 10^−4^–10.26 × 10^−4^)] than in those with only MDD [4.941 × 10^−4^ (3.652 × 10^−4^–6.225 × 10^−4^)] in univariate (p = 0.031) and multivariate (adjusted p = 0.035) analyses. A significant difference in Quinolinic acid levels was found between patients with MDD and T2DM [9.588 × 10^−5^ (6.642 × 10^−5^–1.368 × 10^−4^)] and those with only MDD [1.372 × 10^−4^ (1.033 × 10^−4^–1.978 × 10^−4^)] in univariate analysis (p = 0.045) but not in multivariate analysis (adjusted p = 0.36).

**Table 3:** Differences in the metabolites of the kynurenine and serotonin pathways

	MDD without T2DM	MDD with T2DM	p-value	Adjusted p-value	Effect size
Kynurenine pathway Metabolites					
Tryptophan	0.205 [0.167–0.238]	0.193 [0.164–0.204]	0.36	0.15	0.15
* N*-formylkynurenine	3.314 × 10^−5^ [2.830 × 10^−5^–3.913 × 10^−5^]	3.018 × 10^−5^ [2.342 × 10^−5^–4.092 × 10^−5^]	0.69	0.39	0.07
Kynurenine	6.413 × 10^−3^ [4.758 × 10^−3^–7.710 × 10^−3^]	6.832 × 10^−3^ [4.966 × 10^−3^–7.794 × 10^−3^]	0.40	0.59	0.14
Kynurenic acid	N.D	N.D	**–**	**–**	**–**
3-Hydroxykynurenine	6.670 × 10^−5^ [5.740 × 10^−5^–8.336 × 10^−5^]	7.353 × 10^−5^ [4.978 × 10^−5^–11.16 × 10^−5^]	0.62	0.81	0.08
3-Hydroxyanthranilic acid	N.D	N.D	**–**	**–**	**–**
Quinolinic acid	9.588 × 10^−5^ [6.642 × 10^−5^–1.368 × 10^−4^]	1.372 × 10^−4^ [1.033 × 10^−4^–1.978 × 10^−4^]	0.045	0.36	0.32
Picolinic acid	N.D	N.D	–	–	–
Xanthurenic acid	N.D	N.D	–	–	–
Serotonin pathway Metabolites					
Pretonine	5.012 × 10^−5^ [4.045 × 10^−5^–5.293 × 10^−5^]	4.633 × 10^−5^ [4.241 × 10^−5^–5.965 × 10^−5^]	0.89	0.63	0.02
Serotonin	6.565 × 10^−5^ [3.350 × 10^−5^–2.758 × 10^−4^]	1.233 × 10^−4^ [4.593 × 10^−5^–2.756 × 10^−4^]	0.51	0.61	0.11
* N*-Methylserotonin	N.D	N.D	**–**	**–**	**–**

P-values are adjusted for age, sex, and BMI. The p-value was calculated using the Mann–Whitney U test, and the adjusted p-value was determined by multiple regression analysis. Effect sizes were calculated using the Mann–Whitney U test. *BMI* body mass index, *N.D.* not detected, *MDD* major depressive disorder, *T2DM* type 2 diabetes mellitus

**Table 4:** Differences in the metabolites and ratios of the kynurenine and serotonin pathways

	MDD without T2DM	MDD with T2DM	p-value	Adjusted p-value	Effect size
Kynurenine Pathway Ratio					
N-Formylkynurenine/tryptophan	1.594 × 10^−4^ [1.288 × 10^−4^–2.108 × 10^−4^]	1.910 × 10^−4^ [1.278 × 10^−4^–2.150 × 10^−4^]	0.79	0.96	0.04
Kynurenine/tryptophan	0.028 [0.026–0.035]	0.035 [0.031–0.047]	0.083	0.73	0.28
3-Hydroxykynurenine/tryptophan	3.338 × 10^−4^ [2.447 × 10^−4^–4.561 × 10^−4^]	4.468 × 10^−4^ [3.131 × 10^−4^–7.595 × 10^−4^]	0.39	0.49	0.14
3-Hydroxykynurenine/kynurenine	0.011 [0.0092–0.013]	0.011 [0.0086–0.015]	0.86	0.47	0.03
Quinolinic acid/tryptophan	4.941 × 10^−4^ [3.652 × 10^−4^–6.225 × 10^−4^]	7.093 × 10^−4^ [5.442 × 10^−4^–10.26 × 10^−4^]	0.031	0.035	0.34
Quinolinic acid/kynurenine	0.017 [0.013–0.019]	0.019 [0.018–0.025]	0.064	0.059	0.30
Serotonin Pathway Ratio					
Pretonine/tryptophan	2.460 × 10^–4^ [1.862 × 10^–4^–3.187 × 10^–4^]	2.800 × 10^–4^ [2.032 × 10^–4^–3.058 × 10^–4^]	0.91	0.68	0.02
Serotonin/tryptophan	4.442 × 10^−4^ [1.526 × 10^−4^–1.570 × 10^−3^]	8.074 × 10^−4^ [3.109 × 10^−4^–1.110 × 10^−3^]	0.30	0.50	0.17
Serotonin/pretonine	1.398 [0.649–1.398]	3.769 [1.182–7.168]	0.13	0.67	0.24

P-values are adjusted for age, sex, and BMI. The p-value was calculated using the Mann–Whitney U test, and the adjusted p-value was determined by multiple regression analysis. Effect sizes were calculated using the Mann–Whitney U test. *BMI* body mass index, *N.D.* not detected, *MDD* major depressive disorder, *T2DM* type 2 diabetes mellitus

### Relationship between inflammatory cytokine levels and metabolites of the kynurenine and serotonin pathways in both groups

**Fig. 3:**
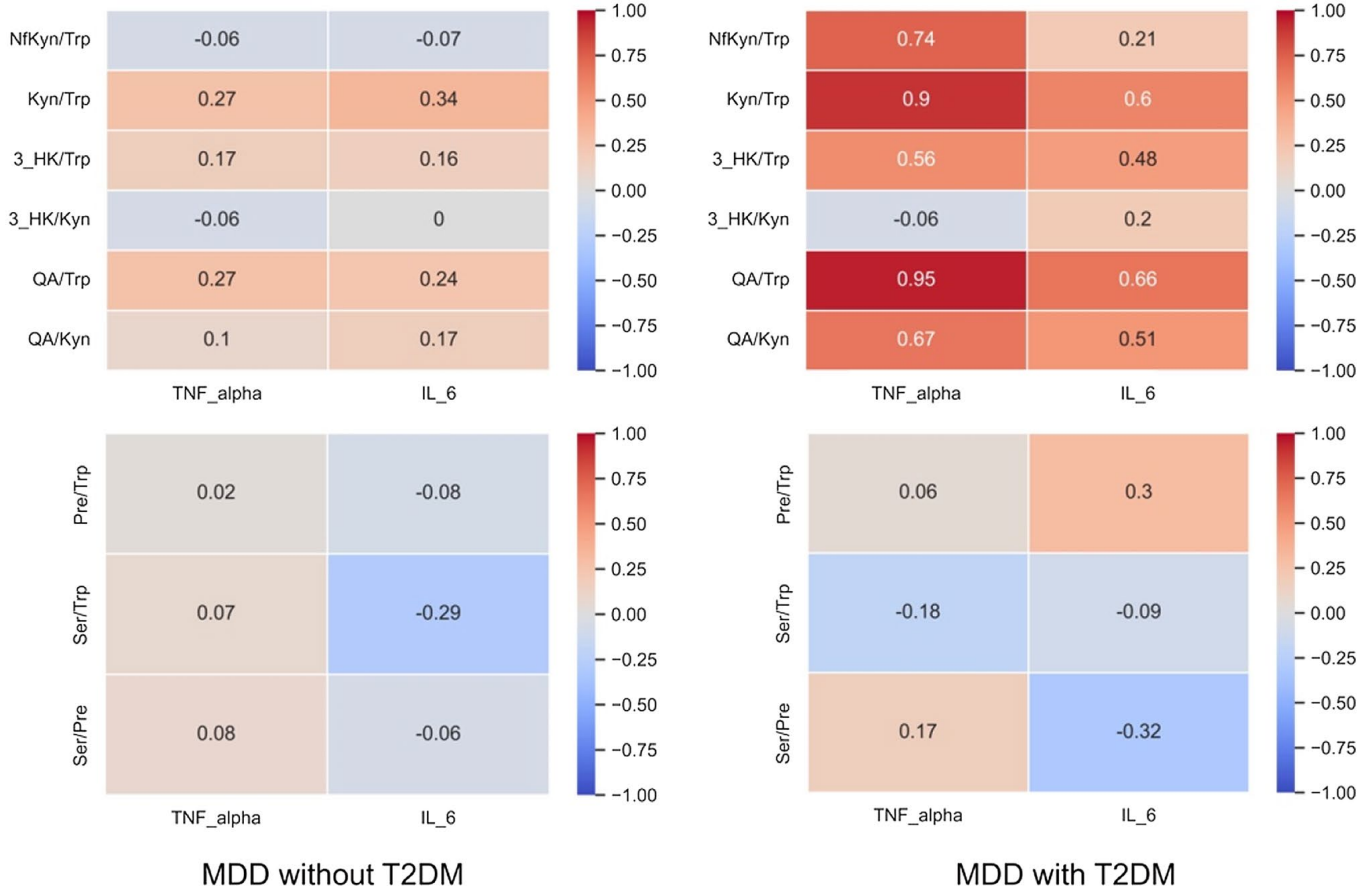
Relationship between inflammatory cytokines and the ratio of kynurenine and serotonin pathway metabolites. We show the relationship between inflammatory cytokines and ratio of kynurenine and serotonin pathway metabolites. The ratio of kynurenine metabolites in MDD with T2DM shows a more positive relationship with inflammatory cytokines, especially TNF-α, than that in MDD without T2D. The relationship is based on Spearman’s rank correlation coefficient. *MDD* major depressive disorder, *NfKyn* N-formylkynurenine, *Pre* pretonine, *QA* quinolinic acid, *Ser* serotonin, *T2DM* type 2 diabetes mellitus, *Trp* tryptophan, *3-HK* 3-hydroxykynurenine

We showed the relationship between inflammatory cytokine levels and the metabolites of the kynurenine and serotonin pathways in patients with MDD and T2DM and those with only MDD, as shown in Online Resources 1 and 2. TNF-α levels indicated statistically significant correlations with tryptophan, kynurenine, 3-hydroxykynurenine/tryptophan, and serotonin levels in patients with MDD without T2DM (β =  − 0.603, adjusted p = 0.032; β = 0.718, adjusted p = 0.002; β = 0.608, adjusted p = 0.003; β = 0.450, adjusted p = 0.049, respectively). In patients with MDD and T2DM, TNF-α levels were significantly correlated with tryptophan, quinolinic acid, kynurenine/tryptophan, quinolinic acid/tryptophan, and quinolinic acid/kynurenine levels (β =  − 0.695, adjusted p = 0.030; β = 0.898, adjusted p = 0.020; β = 0.934, adjusted p = 0.012; β = 0.951, adjusted p < 0.001; β = 0.834, adjusted p = 0.002, respectively). IL-6 levels showed a statistically significant relationship with kynurenine levels in patients with MDD without T2DM (β = 0.520, adjusted p = 0.033). In patients with MDD and T2DM, IL-6 levels were not correlated with the metabolites. The relationship between inflammatory cytokines and the ratio of kynurenine and serotonin pathway metabolites in MDD with and without T2DM (heatmap) in shown in Fig. 3. MDD with T2DM indicated stronger positive relationships between the ratio of kynurenine pathway metabolites and inflammatory cytokines, especially TNF-α, than MDD without T2DM.

### Effect modification of inflammatory cytokine levels on the metabolites of the kynurenine and serotonin pathways in both groups)

**Fig. 4:**
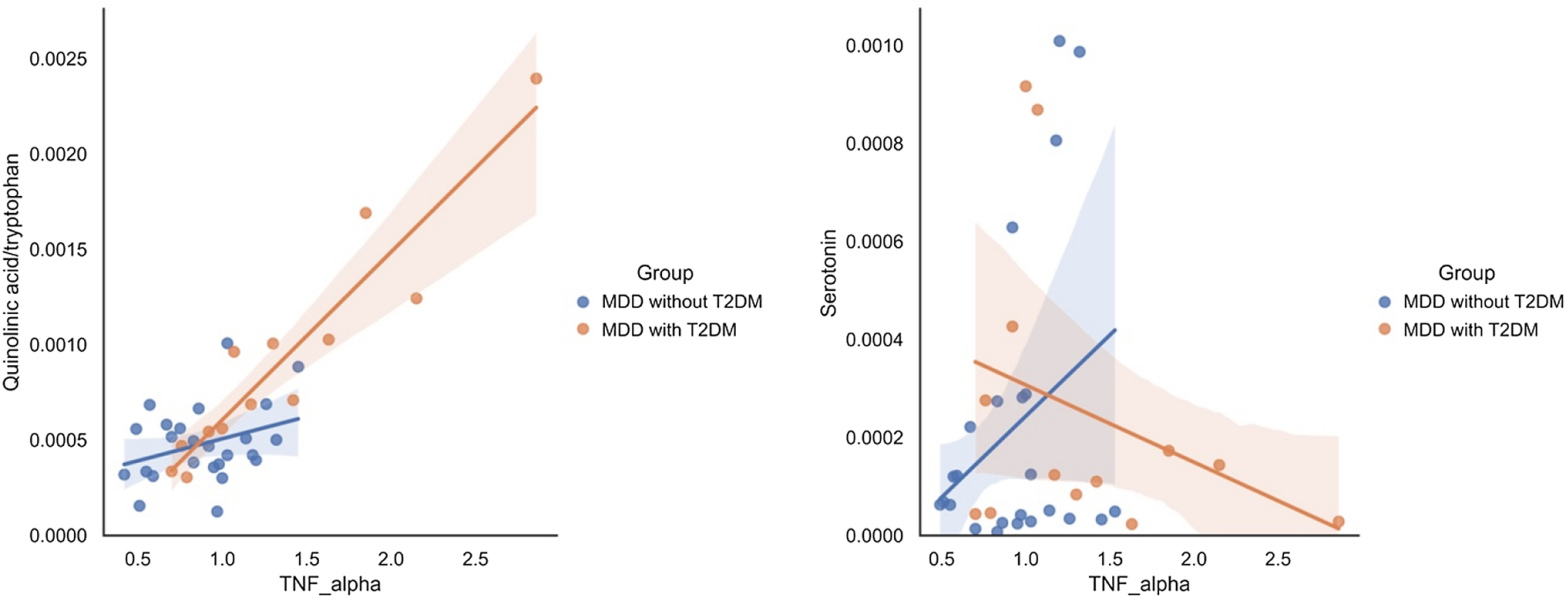
Effect modification (interaction) of inflammatory cytokines on the metabolites of the kynurenine pathway in patients with MDD and T2DM and those with only MDD. TNF-α had a statistically significant effect modification on quinolinic acid/tryptophan and serotonin in patients with MDD and T2DM and those with only MDD (β = 1.029, adjusted p < 0.001; β =  − 1.444, adjusted p = 0.047, respectively). This interaction was measured by comparing the differences in the slopes of each regression line. *TNF-α* tumor necrosis factor-α (pg/mL), serotonin (relative area), *MDD* major depressive disorder, *T2DM* type 2 diabetes mellitus

The effect modification (interaction) of inflammatory cytokine levels on the metabolites of the kynurenine and serotonin pathways in patients with MDD and T2DM and those with only MDD is shown in Table 5. A scatterplot and regression line are shown in Fig. 4. TNF-α levels showed a statistically significant effect modification to quinolinic acid/tryptophan and serotonin levels in patients with MDD and T2DM and those with only MDD (β = 1.029, adjusted p < 0.001; β =  − 1.444, adjusted p = 0.047, respectively). IL-6 levels did not show effect modification. All results of the analysis are shown in Online Resource 3.

**Table 5:** Effect modification of inflammatory cytokines on the metabolites of the kynurenine and serotonin pathways in both groups (interaction analysis)

	Standardized coefficient (β)	Coefficient (B)	95% confidence interval	Standard error	t-value	Adjusted p-value
Group #^a^ TNF-α						
Quinolinic acid/tryptophan	1.029	5.930 × 10^−4^	2.496 × 10^−4^– 9.378 × 10^−4^	1.685 × 10^−4^	3.52	< 0.001
Serotonin	− 1.444	− 5.880 × 10^−4^	− 1.168 × 10^−3^– − 8.322 × 10^−6^	2.835 × 10^−4^	− 2.07	0.047

^a^(#) indicates interaction analysis (i.e., Group # TNF-α shows interaction analysis between TNF-α and MDD with and without T2DM). P-values are adjusted for age, sex, and BMI. The adjusted p-value was determined by multiple regression analysis. *MDD* major depressive disorder, *T2DM* type 2 diabetes mellitus, *TNF-α* tumor necrosis factor-α

### Relationship between inflammatory cytokine levels and metabolites of the kynurenine and serotonin pathways and clinical data in both groups

The relationship between inflammatory cytokine levels and metabolites of the kynurenine and serotonin pathways and clinical data (HAMD scores and HbA1c levels) of patients with comorbid MDD and T2DM and those with only MDD are shown in Online Resources 4 and 5. TNF-α and IL-6 showed no relationship with HAMD scores and HbA1c levels in both groups. In patients with MDD without T2DM, kynurenine and 3-hydroxykynurenine showed a statistically significant relationship with HAMD scores (β =  − 0.496, adjusted p = 0.042; β =  − 0.430, adjusted p = 0.038, respectively). Metabolites of the kynurenine and serotonin pathways showed no relationship with HbA1c levels in both groups.

## Discussion

In this study, we mainly examined the relational and differential effects of inflammatory cytokine levels on the metabolites of the kynurenine and serotonin pathways in patients with comorbid MDD and T2DM and those with only MDD. TNF-α levels and the ratio of quinolinic acid to tryptophan were significantly higher in MDD with T2DM. The serum of patients with MDD and T2DM indicated a stronger positive relationship between the ratio of kynurenine pathway metabolites and inflammatory cytokines, especially TNF-α. TNF-α had an effect modification (interaction) on the quinolinic acid/tryptophan ratio and serotonin levels in patients with MDD, depending on the presence or absence of T2DM. To the best of our best knowledge, this is the first study to show that, in the presence of T2DM, TNF-α causes an effect modification on the kynurenine and serotonin pathways in patients with MDD.

Chronic low-grade inflammation is a common feature of patients with MDD and T2DM [12, 29, 30]. Chronic stress induces immune dysregulation, either directly or indirectly, via the hypothalamic–pituitary–adrenal axis and sympathetic nervous system and increases the production of inflammatory cytokines [5].

The activation of inflammatory cytokines increases the shift in tryptophan metabolic activity from the serotonin to the kynurenine pathway, resulting in a relative decrease in serotonin synthesis [31]. Therefore, decreased tryptophan, kynurenine, and kynurenic acid levels and increased quinolinic acid levels have been reported in MDD; however, there is significant disagreement among studies regarding this [15]. Kynurenine is also associated with the onset of diabetes, and kynurenic acid has antidiabetic properties [10]. N-methyl-D-aspartate (NMDA) receptors are also expressed in pancreatic beta cells, and excess quinolinic acid may be a driver of metabolic diseases through persistent activation of NMDA receptors, resulting in the dysfunction of these cells [32, 33].

In this study, TNF-α levels were significantly higher in patients with MDD and T2DM than in those with only MDD (Fig. 2). TNF-α is a representative inflammatory cytokine that shunts tryptophan into the kynurenine pathway by activating TDO/IDO (Fig. 1) [34]. A previous systematic review and meta-analysis showed no difference in TNF-α levels between patients with MDD and healthy controls [35]. Conversely, patients with treatment-resistant MDD have higher TNF-α levels than healthy controls [36]. TNF-α was associated with treatment resistance through increased expression and activity of the monoamine transporter, which is a target of antidepressants, decreased expression of glutamate transporters, and inhibition of neurogenesis [37]. Prolonged hyperglycemia also shows dysregulation of TNF-α due to associated metabolic abnormalities [38]. TNF-α is also one of the most important inflammatory mediators that correlates with insulin resistance and is critically involved in T2DM pathogenesis [39, 40].

TNF-α expression was significantly correlated with several metabolites of the kynurenine pathway and their ratios in patients with MDD and T2DM and those with only MDD, whereas IL-6 was only positively correlated with kynurenine in patients with MDD without T2DM. Compared with IL-6, TNF-α also showed higher correlation coefficients overall for the ratio of kynurenine pathway metabolites in MDD with T2DM (Fig. 3). We have previously reported that kynurenine/tryptophan levels are strongly correlated with TNF-α levels, but not with IL-6 levels, in a study of chronic-phase schizophrenia [41]. Thus, our results suggest that TNF-α can be a more potent activator of kynurenine pathway shunt formation than IL-6, especially in MDD with T2DM. To date, there have been few reports on the differences in the relationship between types of inflammatory cytokines and the kynurenine and serotonin pathways in MDD, and further studies are required.

The most distinct and attractive aspect of this study is the finding that TNF-α has an effect modification (interaction) on the quinolinic acid/tryptophan ratio and serotonin levels depending on the presence of T2DM (Fig. 4). Quinolinic acid is an NMDA receptor agonist, and its activating effects suggest that inflammation is an important mechanistic pathway for its depressant effects [8, 42]. Quinolinic acid has similar potency to glutamate at NMDA receptors; however, its reuptake efficiency is lower, and it remains in the synaptic cleft for longer [43]. Thus, quinolinic acid is likely to alter neuroplasticity via NMDA receptors, and sufficient concentrations of quinolinic acid can contribute to direct neuroexcitotoxicity [44–46]. MDD with T2DM is less responsive to treatment than MDD without T2DM; however, the reason for this is unknown [23]. Although serotonin selective receptor inhibitors cause presynaptic reuptake of serotonin at the serotonin transporter and increase serotonin in the postsynaptic membrane [47], some of their therapeutic effects are reported as anti-inflammatory ones [48]. Antidepressant effects of anti-inflammatory drugs have long been noted [31, 49]. In particular, ketamine shows anti-inflammatory and inflammation-modulating effects in the central and peripheral systems and has been actively studied in recent years for its effects on a subgroup of patients with treatment-resistant MDD, particularly those with high levels of inflammation [50, 51]. The results of this study showing an effect modification by TNF-α on kynurenine and serotonin pathways in comorbid T2DM suggest that MDD with T2DM has more of an inflammatory depressive component.

Taken together, our findings suggest that MDD with T2DM is associated with higher chronic shunt enhancement and activation of the kynurenine pathway by inflammatory cytokines than MDD without T2DM. The resultant increase in the neuroexcitation of NMDA receptors by quinolinic acid induces irreversible changes in the brain, which may be associated with refractoriness to conventional antidepressant drugs. Our findings suggest that the use of a combination of antidepressants and anti-inflammatory drugs for MDD with T2DM may be favorable, giving this study clinical utility.

## Limitations

This study had some limitations. First, all patients were Japanese, and the severity of MDD was mild to moderate; therefore, the findings may not be generalizable to patients with more severe MDD phenotypes. Second, our small sample size could have lowered the statistical power because of type 2 errors. However, because interaction analysis generally tends to decrease power, this study showed a statistically significant difference despite the small sample size. Third, conversely, type 1 errors owing to multiple testing must also be considered. For example, the relationship between serotonin and TNF-α levels showed greater variability than that between quinolinic acid/tryptophan levels and should be interpreted with caution. Fourth, drugs for MDD and T2DM may also influence these metabolites. Fifth, we used non-fasting and time-randomized samples, which may have affected the results. Sixth, this was a one-point observational study, and changes over time owing to treatment and other factors are unknown. We selected basic demographic data of age, sex, and BMI as covariates in our multivariate analysis because of the small sample size. However, this was an observational study and not a randomized controlled trial; unobserved confounding and other factors may have influenced our result. Finally, kynurenic, 3-hydroxyanthranilic, picolinic, xanthurenic and acids, as well as N-methylserotonin, were difficult to analyze because of technical problems, either in terms of detection thresholds or the number of detections. The effects of other pro-inflammatory cytokines, such as IL-1β and IFN-β, and inhibitory cytokines, such as IL-10, were not evaluated. To address these issues, it is desirable to further increase the number of specimens and measurements and conduct a prospective longitudinal study of first-episode, drug-naive patients with MDD.

## Conclusions

TNF-α levels were higher in patients with MDD and T2DM than in those with only MDD, and TNF-α had an effect modification on the quinolinic acid/tryptophan ratio and serotonin in patients depending on the presence of T2DM. These findings suggest that MDD with T2DM has enhanced activation of the kynurenine pathway by inflammatory cytokines compared to MDD without T2DM, and administration of a combination of antidepressants and anti-inflammatory drugs is possibly more effective in patients with comorbid MDD and T2DM.

## Supplementary Information

Below is the link to the electronic supplementary material.

Supplementary file1 (DOCX 24 KB)

Supplementary file2 (DOCX 23 KB)

Supplementary file3 (DOCX 19 KB)

Supplementary file4 (DOCX 23 KB)

Supplementary file5 (DOCX 23 KB)

## Data Availability

The data that support the findings of this study are available upon reasonable request from the corresponding author.
