# Supplementary material for: Effect modification of tumor necrosis factor-α on the kynurenine and serotonin pathways in major depressive disorder on type 2 diabetes mellitus
Source: Eur Arch Psychiatry Clin Neurosci. 2023 Nov 22;274(7):1697–707. doi: 10.1007/s00406-023-01713-8 (PMC11422469; doi:10.1007/s00406-023-01713-8)
Supplement: Supplementary file 1 — Supplementary file1 (DOCX 24 KB) [file 406_2023_1713_MOESM1_ESM.docx]

*European Archives of Psychiatry and Clinical Neuroscience*

**Effect modification of tumor necrosis factor-α on the kynurenine and serotonin pathways in major depressive disorder on type 2 diabetes mellitus**

Naomichi Okamoto, Takashi Hoshikawa, Yuichi Honma, Enkhmurun Chibaatar, Atsuko Ikenouchi, Masaru Harada, and Reiji Yoshimura

Corresponding author: Naomichi Okamoto

Department of Psychiatry, University of Occupational and Environmental Health, Fukuoka, Japan

E-mail address: [nokamoto@med.uoeh-u.ac.jp](mailto:nokamoto@med.uoeh-u.ac.jp)

**Online Resource 1** **Relationship between the metabolites of the kynurenine and serotonin pathways and inflammatory cytokines in patients with MDD without T2DM**

|  | Univariate analysis | | Multivariate analysis | | | | | |
| --- | --- | --- | --- | --- | --- | --- | --- | --- |
|  | Spearman  (r) | p-value | Standardized coefficient  (β) | Coefficient  (B) | 95% confidence interval | Standard error | t-value | Adjusted  p-value |
| *TNF-α* |  |  |  |  |  |  |  |  |
| Tryptophan | −0.024 | 0.90 | −0.603 | −4.752 | −9.063–−0.442 | 2.072687 | −2.29 | 0.032 |
| Kynurenine | 0.480 | 0.012 | 0.718 | 159.1 | 67.36–250.9 | 44.012 | 3.61 | 0.002 |
| Quinolinic acid | 0.311 | 0.13 | 0.374 | 2199 | 1115–5558 | 1604 | 1.37 | 0.18 |
| Kynurenine/tryptophan | 0.277 | 0.18 | 0.264 | 10.51 | −8.773–  29.80 | 9.216 | 1.14 | 0.26 |
| 3-Hydroxykynurenine/tryptophan | 0.173 | 0.38 | 0.608 | 855.1 | 329.8–  1380 | 252.6 | 3.39 | 0.003 |
| Quinolinic acid/tryptophan | 0.253 | 0.22 | 0.491 | 686.6 | −99.62–  1473 | 375.6 | 1.83 | 0.083 |
| Quinolinic acid/kynurenine | 0.108 | 0.60 | 0.230 | 11.36 | −13.51–  36.24 | 11.88 | 0.96 | 0.35 |
| Serotonin | 0.120 | 0.57 | 0.450 | 430.73 | 2.377–859.0 | 203.8 | 2.11 | 0.049 |
| *IL-6* |  |  |  |  |  |  |  |  |
| Kynurenine | 0.440 | 0.024 | 0.520 | 445.2 | 40.96–  849.5 | 193.8 | 2.30 | 0.033 |

P-values are adjusted for age, sex, and BMI. The p-value was calculated using Spearman’s rank correlation coefficient, and the adjusted p-value was calculated using multiple regression analysis. MDD, major depressive disorder; T2DM, type 2 diabetes mellitus; BMI, body mass index; TNF-α, tumor necrosis factor-α; IL-6, interleukin-6.
