# Supplementary material for: Effect modification of tumor necrosis factor-α on the kynurenine and serotonin pathways in major depressive disorder on type 2 diabetes mellitus
Source: Eur Arch Psychiatry Clin Neurosci. 2023 Nov 22;274(7):1697–707. doi: 10.1007/s00406-023-01713-8 (PMC11422469; doi:10.1007/s00406-023-01713-8)
Supplement: Supplementary file 2 — Supplementary file2 (DOCX 23 KB) [file 406_2023_1713_MOESM2_ESM.docx]

*European Archives of Psychiatry and Clinical Neuroscience*

**Effect modification of tumor necrosis factor-α on the kynurenine and serotonin pathways in major depressive disorder on type 2 diabetes mellitus**

Naomichi Okamoto, Takashi Hoshikawa, Yuichi Honma, Enkhmurun Chibaatar, Atsuko Ikenouchi, Masaru Harada, and Reiji Yoshimura

Corresponding author: Naomichi Okamoto

Department of Psychiatry, University of Occupational and Environmental Health, Fukuoka, Japan

E-mail address: [nokamoto@med.uoeh-u.ac.jp](mailto:nokamoto@med.uoeh-u.ac.jp)

**Online Resource 2** **Relationship between metabolites of the kynurenine and serotonin pathways and inflammatory cytokines in patients with MDD and T2DM**

|  | Univariate analysis | | Multivariate analysis | | | | | |
| --- | --- | --- | --- | --- | --- | --- | --- | --- |
|  | Spearman  (r) | p-value | Standardized coefficient  (β) | Coefficient  (B) | 95% confidence interval | Standard error | t-value | Adjusted  p-value |
| *TNF-α* |  |  |  |  |  |  |  |  |
| Tryptophan | −0.62 | 0.026 | −0.695 | −10.81 | −20.27–−1.349 | 4.103 | −2.63 | 0.030 |
| Kynurenine | 0.43 | 0.14 | 0.198 | 45.28 | −192.3–  282.9 | 103.0 | 0.44 | 0.67 |
| Quinolinic acid | 0.84 | < 0.001 | 0.898 | 7038 | 1408–  12668 | 2441 | 2.88 | 0.020 |
| Kynurenine/tryptophan | 0.90 | < 0.001 | 0.934 | 42.67 | 12.14–  73.21 | 13.24 | 3.22 | 0.012 |
| 3-Hydroxykynurenine/tryptophan | 0.56 | 0.049 | 0.384 | 955.6 | −893.7–  2805 | 802.0 | 1.19 | 0.26 |
| Quinolinic acid/tryptophan | 0.95 | < 0.001 | 0.951 | 1013 | 669.3–  1358 | 149.4 | 6.79 | < 0.001 |
| Quinolinic acid/kynurenine | 0.67 | 0.014 | 0.834 | 65.38 | 31.58–  99.18 | 14.65 | 4.46 | 0.002 |
| Serotonin | −0.25 | 0.39 | −0.310 | −638.8 | −2176–  898.7 | 666.7 | −0.96 | 0.36 |
| *IL-6* |  |  |  |  |  |  |  |  |
| Kynurenine | 0.22 | 0.47 | −0.421 | −469.3 | −1487–  549.3 | 441.7 | −1.06 | 0.31 |

P-values are adjusted for age, sex, and BMI. The p-value was calculated using Spearman’s rank correlation coefficient, and the adjusted p-value was calculated using multiple regression analysis. MDD, major depressive disorder; T2DM, type 2 diabetes mellitus; BMI, body mass index; TNF-α, tumor necrosis factor-α; IL-6, interleukin-6.
