# Supplementary material for: Effect modification of tumor necrosis factor-α on the kynurenine and serotonin pathways in major depressive disorder on type 2 diabetes mellitus
Source: Eur Arch Psychiatry Clin Neurosci. 2023 Nov 22;274(7):1697–707. doi: 10.1007/s00406-023-01713-8 (PMC11422469; doi:10.1007/s00406-023-01713-8)
Supplement: Supplementary file 3 — Supplementary file3 (DOCX 19 KB) [file 406_2023_1713_MOESM3_ESM.docx]

*European Archives of Psychiatry and Clinical Neuroscience*

**Effect modification of tumor necrosis factor-α on the kynurenine and serotonin pathways in major depressive disorder on type 2 diabetes mellitus**

Naomichi Okamoto, Takashi Hoshikawa, Yuichi Honma, Enkhmurun Chibaatar, Atsuko Ikenouchi, Masaru Harada, and Reiji Yoshimura

Corresponding author: Naomichi Okamoto

Department of Psychiatry, University of Occupational and Environmental Health, Fukuoka, Japan

E-mail address: [nokamoto@med.uoeh-u.ac.jp](mailto:nokamoto@med.uoeh-u.ac.jp)

**Online Resource 3 Effect modification of inflammatory cytokine levels on metabolites in the kynurenine and serotonin pathways in patients with MDD with and without T2DM (interaction analysis)**

|  | Standardized coefficient  (β) | Coefficient  (B) | 95% Confidence interval | Standard error | t-value | Adjusted  p-value |
| --- | --- | --- | --- | --- | --- | --- |
| *Group # ^a^ TNF-α* |  |  |  |  |  |  |
| Tryptophan | 0.254 | 1.627 × 10^-2^ | –3.824 × 10^-2^  – 7.079 × 10^-2^ | 2.676 × 10^-2^ | 0.61 | 0.54 |
| Kynurenine | –0.549 | –1.598 × 10^-3^ | –4.239 × 10^-3^– 1.042 × 10^-3^ | 1.294 × 10^-3^ | –1.23 | 0.22 |
| Quinolinic acid | 0.288 | 2.570 × 10^-5^ | –6.140 × 10^-5^  –1.127 × 10^-4^ | 4.260 × 10^-5^ | 0.60 | 0.55 |
| Kynurenine/tryptophan | 0.445 | 6.400 × 10^-3^ | –7.359 × 10^-3^  –2.016 × 10^-2^ | 6.737 × 10^-3^ | 0.95 | 0.35 |
| 3-Hydroxykynurenine/tryptophan | –0.731 | –2.706 × 10^-4^ | –6.065 × 10^-4^–  6.540 × 10^-4^ | 1.649 × 10^-4^ | –1.64 | 0.11 |
| Quinolinic acid/tryptophan | 1.029 | 5.937 × 10^-4^ | 2.496 × 10^-4^ –  9.378 × 10^-4^ | 1.685 × 10^-4^ | 3.52 | < 0.001 |
| Quinolinic acid/kynurenine | 0.696 | 6.64 × 10^-3^ | –2.878 × 10^-3^  –1.617 × 10^-2^ | 4.665 × 10^-3^ | 1.43 | 0.16 |
| Serotonin | –1.444 | –5.880 × 10^-4^ | –1.168 × 10^-3^ –  –8.322 × 10^-6^ | 2.835 × 10^-4^ | –2.07 | 0.047 |
| *Group # IL-6* |  |  |  |  |  |  |
| Kynurenine | –0.529 | –4.767 × 10^-4^ | –1.167 × 10^-3^  –2.145 × 10^-4^ | 3.389 × 10^-4^ | –1.41 | 0.16 |

^a^(#) indicates interaction analysis (i.e., Group # TNF-α shows interaction analysis between TNF-α and MDD with and without T2DM). P-values are adjusted for age, sex, and BMI. The p-value was calculated using Spearman’s rank correlation coefficient, and the adjusted p-value was calculated using multiple regression analysis. MDD, major depressive disorder; T2DM, type 2 diabetes mellitus; BMI, body mass index; TNF-α, tumor necrosis factor-α; IL-6, interleukin-6.
