# Supplementary material for: Effect modification of tumor necrosis factor-α on the kynurenine and serotonin pathways in major depressive disorder on type 2 diabetes mellitus
Source: Eur Arch Psychiatry Clin Neurosci. 2023 Nov 22;274(7):1697–707. doi: 10.1007/s00406-023-01713-8 (PMC11422469; doi:10.1007/s00406-023-01713-8)
Supplement: Supplementary file 4 — Supplementary file4 (DOCX 23 KB) [file 406_2023_1713_MOESM4_ESM.docx]

*European Archives of Psychiatry and Clinical Neuroscience*

**Effect modification of tumor necrosis factor-α on the kynurenine and serotonin pathways in major depressive disorder on type 2 diabetes mellitus**

Naomichi Okamoto, Takashi Hoshikawa, Yuichi Honma, Enkhmurun Chibaatar, Atsuko Ikenouchi, Masaru Harada, and Reiji Yoshimura

Corresponding author: Naomichi Okamoto

Department of Psychiatry, University of Occupational and Environmental Health, Fukuoka, Japan

E-mail address: [nokamoto@med.uoeh-u.ac.jp](mailto:nokamoto@med.uoeh-u.ac.jp)

**Online Resource 5 Relationship between inflammatory cytokines and clinical data (HAMD scores and HbA1c levels) in patients with T2DM**

|  | Univariate analysis | | Multivariate analysis | | | | | |
| --- | --- | --- | --- | --- | --- | --- | --- | --- |
|  | Spearman  (r) | p-value | Standardized coefficient  (β) | Coefficient  (B) | 95% confidence interval | Standard error | t-value | Adjusted  p-value |
| *HAMD* |  |  |  |  |  |  |  |  |
| TNF-α | 0.113 | 0.71 | 0.004 | 0.051 | −8.893−8.996 | 3.879 | 0.01 | 0.99 |
| IL-6 | 0.14 | 0.64 | 0.439 | 0.943 | −0.8784−2.764 | 0.789 | 1.19 | 0.26 |
| Tryptophan | −0.329 | 0.27 | −0.060 | −9.936 | −151.5−131.7 | 61.42 | −0.16 | 0.87 |
| N-formylkynurenine | −0.179 | 0.38 | 0.182 | 107595 | −392567−607758 | 216896 | 0.50 | 0.63 |
| Kynurenine | 0.077 | 0.80 | 0.377 | 902 | −1632−3436 | 1099 | 0.82 | 0.43 |
| 3-Hydroxykynurenine | 0.260 | 0.39 | 0.477 | 59023 | −28678−146725 | 38031 | 1.55 | 0.15 |
| Quinolinic acid | −0.083 | 0.78 | −0.078 | −6416 | −94464−81632 | 38182 | −0.17 | 0.87 |
| Pretonine | −0.032 | 0.92 | 0.004 | 1779 | −592602−596161 | 242911 | 0.01 | 0.99 |
| Serotonin | −0.583 | 0.036 | −0.497 | −10744 | −26254−4765 | 6725 | −1.60 | 0.14 |
| *HbA1c* |  |  |  |  |  |  |  |  |
| TNF-α | −0.165 | 0.62 | −0.206 | −0.396 | −2.346−1.554 | 0.797 | −0.50 | 0.63 |
| IL-6 | −0.339 | 0.30 | −0.352 | −0.123 | −0.577−0.331 | 0.185 | −0.66 | 0.53 |
| Tryptophan | 0.073 | 0.83 | 0.092 | 2.523 | −25.93−30.97 | 11.62 | 0.22 | 0.83 |
| N-formylkynurenine | 0.183 | 0.58 | 0.293 | 33159 | −74055−140374 | 43816 | 0.76 | 0.47 |
| Kynurenine | −0.036 | 0.91 | 0.195 | 122.6 | −672.9−918.3 | 325.1 | 0.38 | 0.71 |
| 3-Hydroxykynurenine | −0.100 | 0.76 | −0.037 | −845.6 | −23256−21565 | 9158 | −0.09 | 0.92 |
| Quinolinic acid | −0.495 | 0.12 | −0.587 | −13218 | −41635−15199 | 11613 | −1.14 | 0.29 |
| Pretonine | −0.075 | 0.84 | −1.017 | −93509 | −220673−33653 | 45800 | −2.04 | 0.11 |
| Serotonin | −0.082 | 0.89 | −0.578 | −2037 | −5138−1063 | 1267 | −1.61 | 0.15 |

P-values are adjusted for age, sex, and BMI. The p-value was calculated using Spearman’s rank correlation coefficient, and the adjusted p-value was calculated using multiple regression analysis. HAMD, Hamilton Depression Rating Scale; HbA1c, hemoglobin A1c; T2DM, type 2 diabetes mellitus; BMI; body mass index; TNF-α, tumor necrosis factor-α; IL-6, interleukin-6.
