# Supplementary material for: Effect modification of tumor necrosis factor-α on the kynurenine and serotonin pathways in major depressive disorder on type 2 diabetes mellitus
Source: Eur Arch Psychiatry Clin Neurosci. 2023 Nov 22;274(7):1697–707. doi: 10.1007/s00406-023-01713-8 (PMC11422469; doi:10.1007/s00406-023-01713-8)
Supplement: Supplementary file 5 — Supplementary file5 (DOCX 23 KB) [file 406_2023_1713_MOESM5_ESM.docx]

*European Archives of Psychiatry and Clinical Neuroscience*

**Effect modification of tumor necrosis factor-α on the kynurenine and serotonin pathways in major depressive disorder on type 2 diabetes mellitus**

Naomichi Okamoto, Takashi Hoshikawa, Yuichi Honma, Enkhmurun Chibaatar, Atsuko Ikenouchi, Masaru Harada, and Reiji Yoshimura

Corresponding author: Naomichi Okamoto

Department of Psychiatry, University of Occupational and Environmental Health, Fukuoka, Japan

E-mail address: [nokamoto@med.uoeh-u.ac.jp](mailto:nokamoto@med.uoeh-u.ac.jp)

**Online Resource 4 Relationship between inflammatory cytokines and clinical data (HAMD and HbA1c) in patients without T2DM**

|  | Univariate analysis | | Multivariate analysis | | | | | |
| --- | --- | --- | --- | --- | --- | --- | --- | --- |
|  | Spearman  (r) | p-value | Standardized coefficient  (β) | Coefficient  (B) | 95% confidence interval | Standard error | t-value | Adjusted  p-value |
| *HAMD* |  |  |  |  |  |  |  |  |
| TNF-α | −0.027 | 0.89 | −0.115 | −1.972 | −9.465–5.520 | 3.603 | −0.55 | 0.59 |
| IL-6 | −0.138 | 0.49 | −0.032 | −0.149 | −2.213−1.915 | 0.992 | −0.15 | 0.88 |
| Tryptophan | 0.072 | 0.72 | 0.200 | −9.936 | −52.37−106.0 | 38.09 | 0.70 | 0.48 |
| N-formylkynurenine | 0.135 | 0.65 | −0.151 | −87042 | −355519−181434 | 128706 | −0.68 | 0.50 |
| Kynurenine | −0.406 | 0.039 | −0.496 | −1939 | −3806−72.77 | 895 | −2.17 | 0.042 |
| 3-Hydroxykynurenine | −0.401 | 0.038 | −0.430 | −81652 | −158346−4958 | 36878 | −2.21 | 0.038 |
| Quinolinic acid | −0.224 | 0.28 | −0.284 | −39954 | −128846−48937 | 42470 | −0.94 | 0.35 |
| Pretonine | 0.174 | 0.38 | 0.025 | 5291 | −89238−99821 | 45455 | 0.12 | 0.90 |
| Serotonin | 0.144 | 0.50 | 0.152 | 3035 | −7006−13077 | 4779 | 0.64 | 0.53 |
| *HbA1c* |  |  |  |  |  |  |  |  |
| TNF-α | −0.124 | 0.54 | −0.331 | −0.392 | −0.791−0.005 | 0.191 | −2.06 | 0.053 |
| IL-6 | −0.051 | 0.80 | −0.258 | −0.123 | −0.19−0.316 | 0.054 | −0.66 | 0.14 |
| Tryptophan | 0.364 | 0.067 | 0.305 | 2.919 | −1.555−7.349 | 2.145 | 1.36 | 0.18 |
| N-formylkynurenine | 0.258 | 0.21 | 0.140 | 4879 | −9802−19562 | 7014 | 0.70 | 0.49 |
| Kynurenine | 0.192 | 0.35 | −0.233 | −64.47 | −181−52.25 | 55.76 | −1.16 | 0.26 |
| 3-Hydroxykynurenine | 0.113 | 0.58 | −0.070 | −954.2 | −6008−4099 | 2422 | −0.39 | 0.69 |
| Quinolinic acid | 0.529 | 0.0078 | 0.312 | 2609 | −2055−7273 | 2220 | 1.18 | 0.25 |
| Pretonine | 0.005 | 0.98 | 0.029 | 419 | −5054−5893 | 2624 | 0.16 | 0.87 |
| Serotonin | −0.276 | 0.19 | 0.116 | 154.8 | −426.7−736.3 | 276.7 | 0.56 | 0.58 |

P-values are adjusted for age, sex, and BMI. The p-value was calculated using Spearman’s rank correlation coefficient, and the adjusted p-value was calculated using multiple regression analysis. HAMD, Hamilton Depression Rating Scale; HbA1c, hemoglobin A1c; BMI; body mass index; T2DM, type 2 diabetes mellitus; TNF-α, tumor necrosis factor-α; IL-6, interleukin-6.
